# Sperm defects in primary ciliary dyskinesia and related causes of male infertility

**DOI:** 10.1007/s00018-019-03389-7

**Published:** 2019-11-28

**Authors:** Anu Sironen, Amelia Shoemark, Mitali Patel, Michael R. Loebinger, Hannah M. Mitchison

**Affiliations:** 1 Genetics and Genomic Medicine, UCL Great Ormond Street Institute of Child Health, University College London, 30 Guilford Street, London, WC1N 1EH UK; 2 Department of Paediatrics, Royal Brompton Hospital, London, UK; 3 School of Medicine, University of Dundee, Dundee, UK; 4 Host Defence Unit, Royal Brompton and Harefield NHS Foundation Trust, London, UK; 5 National Heart and Lung Institute, Imperial College London, London, UK

**Keywords:** PCD, MMAF, Infertility, Cilia, Axoneme, Sperm tail, Motility, Dynein

## Abstract

The core axoneme structure of both the motile cilium and sperm tail has the same ultrastructural 9 + 2 microtubular arrangement. Thus, it can be expected that genetic defects in motile cilia also have an effect on sperm tail formation. However, recent studies in human patients, animal models and model organisms have indicated that there are differences in components of specific structures within the cilia and sperm tail axonemes. Primary ciliary dyskinesia (PCD) is a genetic disease with symptoms caused by malfunction of motile cilia such as chronic nasal discharge, ear, nose and chest infections and pulmonary disease (bronchiectasis). Half of the patients also have situs inversus and in many cases male infertility has been reported. PCD genes have a role in motile cilia biogenesis, structure and function. To date mutations in over 40 genes have been identified cause PCD, but the exact effect of these mutations on spermatogenesis is poorly understood. Furthermore, mutations in several additional axonemal genes have recently been identified to cause a sperm-specific phenotype, termed multiple morphological abnormalities of the sperm flagella (MMAF). In this review, we discuss the association of PCD genes and other axonemal genes with male infertility, drawing particular attention to possible differences between their functions in motile cilia and sperm tails.

## Introduction

**Fig. 1:**
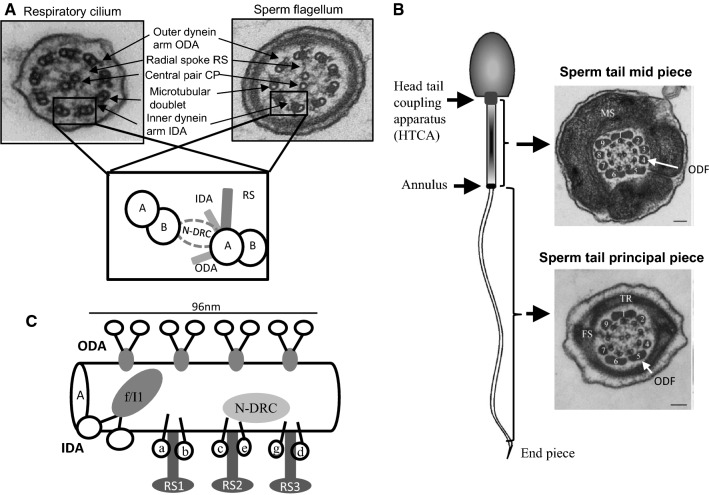
Axonemal structure of respiratory cilia and sperm flagella. **a** The shared electron microscopic axonemal structure of the 9 + 2 motile respiratory cilia and sperm flagellum with major structural features indicated. Outer doublet microtubules are connected to the central pair (CP) by radial spokes (RS) and to each other by nexin links (nexin–dynein regulatory complex, N-DRC). Outer and inner dynein arms (ODA and IDA, respectively) provide the energy for the movement. **b** Accessory structures of the sperm tail. The 9 + 2 axoneme is surrounded by outer dense fibres (ODF) in the mid and principal piece of the sperm tail. Along the mid piece the ODFs are surrounded by mitochondria and along the principal piece the fibrous sheath replaces ODFs 3 and 8 and the transverse ribs (TR) encircle ODFs. The mid-piece and principal piece are separated by the annulus and the tail is connected to the head by the head tail coupling apparatus (HTCA). The formation of sperm tail structures and associated proteins have been previously reviewed [58]. **c** Schematic presentation of the protein complex 96 nm repeat units along the A doublet microtubule. The complex is formed of four identical ODAs, one two-headed IDA (f/I1), six single-headed IDAs (a–e and g), three RS and a single N-DRC

Motile cilia and flagella have a conserved axonemal structure consisting of a ring of nine microtubular doublets and a central pair of microtubules, giving the classical 9 + 2 microtubular arrangement (Fig. 1a). Central pair microtubules C1 and C2 are connected by a bridge-like structure and several projections are docked to C1 and C2 forming the central pair complex (CPC). Each outer doublet is composed of type A and B microtubules and connected by radial spokes (RS) to CPC (Fig. 1a). The force for motility is produced by inner and outer dynein arms (IDA and ODA, respectively), which are carried by the A-type tubule and project toward the B-tubule of the adjacent doublet. IDA and ODA are part of a specific protein complex of 96 nm repeat units, which contains four identical ODAs, one two-headed IDA (f/I1), six single-headed IDAs (a–e and g), three RS and a single nexin–dynein regulatory complex (N-DRC) (Fig. 1c) The N-DRC regulates and coordinates the activity of the dynein arms [43]. The current knowledge of motile cilia ultrastructure and protein content has been previously reviewed by Osinka et al. [96]. The sperm flagellum has an ultrastructurally comparable axonemal structure, but in addition the sperm tail contains accessory structures: the mitochondrial sheath (MS), fibrous sheath (FS) and outer dense fibres (ODFs) (Fig. 1b). These structures are specific to the sperm tail and are required for fertile sperm production providing, e.g. additional rigidity and energy for the movement of the sperm in the female reproductive tract [58]. Sperm tail is connected to the head by the head tail coupling apparatus (HTCA) and annulus forms a diffusion barrier between the mid-piece and principal piece (Fig. 1b). Any alteration in protein functions within these structures can be expected to have a sperm-specific phenotype. The formation of specific sperm tail structures and their associated proteins have been recently reviewed in detail [58]. Furthermore, although the axonemal structure appears to be highly conserved between sperm tail and motile cilia, several studies have suggested that differences exist based on tissue-specific gene and protein expression [1, 2, 6, 19, 63, 130, 135, 137].

Motile cilia malfunction causes primary ciliary dyskinesia (PCD), which is a genetic condition affecting approximately 1:10,000 individuals worldwide [73, 106]. Symptoms frequently start in the neonatal period and include a chronic nasal discharge and wet cough, progressing in childhood to recurrent ear, nose and chest infections and eventual scarring of the lungs in the form of bronchiectasis. The cause of the symptoms is the malfunction of airway motile cilia, which are responsible for mucus clearance in the airways. Motile cilia are also present in the brain, oviduct, Eustachian tube and middle ear, where they function in fluid movement and in the embryonic node during development. Defects in the nodal cilia cause situs inversus approximately in half of PCD patients [7, 131]. PCD is a lifelong condition, the impact of symptoms changing as the patient reaches adulthood. Ear symptoms often attenuate, but bronchiectasis usually progresses and many patients experience fertility problems when trying to start a family [101, 129]. Clinical manifestations and management of PCD have been described in previous publications [36, 73, 106].

Despite extensive and increasing understanding of the spectrum of cilia structural defects in PCD, only few reports exist on the sperm structural defects affecting males with PCD. An early study of the association of male infertility with PCD was done at the Royal Brompton Hospital London in 1994 [86]. Twelve men with PCD had their respiratory cilia and sperm flagella analysed for motility and ultrastructure. Two patients had immotile sperm and one had normal motility with oligospermia (low sperm numbers; Table 1 explains terminology of sperm phenotypic defects). Three of these patients had normal sperm motility and ultrastructure and one had fathered a child. The twelfth patient had slowed sperm with ultrastructural dynein deficiency, but had also reportedly fathered a child. Five had azoospermia (no sperm), which the authors attributed at the time to defects in the ciliated portion of the vas deferens preventing normal transport. However, there are no cilia in the vas deferens, but motile cilia are present in the efferent ductules [20, 142]. Recent studies have shown that these cilia are not required for transport of sperm towards the cauda epididymis, but they agitate the seminal fluid to prevent blockage and to enable reabsorption of the fluid by a rotational swirling motion rather than forward ciliary beat [142]. Thus, the lack of sperm is unlikely to be caused by defects in sperm transport, but may arise from blockage of the efferent ductules. The role of motile cilia in the efferent ductules has not been studied in PCD patients, but mutant mouse models have shown that male infertility can be caused by reduced numbers of motile cilia in the efferent ductules [127, 142]. Furthermore, animal models have also shown that severe spermatid malformations can lead to very low sperm counts in the epididymis, due to sloughing of immature spermatids.

**Table 1:** Forms of male infertility associated with primary ciliary dyskinesia (PCD)

Sperm defect	Phenotype in ejaculate	Alternative name	Associated with PCD
Azoospermia	Absence of spermatozoa	Aspermia	Yes
Teratospermia	Malformed sperm	Teratozoospermia	Yes
Globospermia	Round-headed sperm	Globozoospermia	Not likely
Oligospermia	Low sperm count	Oligozoospermia	Yes
Oligoasthenospermia	Low sperm count associated with low sperm motility	Oligoasthenozoospermia	Most likely
Asthenospermia	Motility defects in the sperm	Asthenozoospermia	Most likely
Asthenoteratospermia	Malformed sperm with motility defects	Asthenoteratozoospermia	Most likely

More recently, a 2017 study reported phenotype–genotype correlations for fertility in 46 male PCD patients, however neither the fertility status of the partner nor the sperm ultrastructure or motility were reported [130]. This study included the cilia structural defects and fertility data available for cases with mutations in 17 different PCD genes, which indicated that the PCD patients more likely to be infertile had cilia showing a loss of inner dynein arms with microtubular disorganization, or a lack of the outer and inner dynein arms. Recent advances in PCD genetics will increasingly enable investigations of the effect of specific mutations on male fertility and prediction of their effect based on the genetic test. However, prior to implementing fertility counselling in PCD clinics, the association of specific mutations with sperm phenotype needs to be elucidated. More comprehensive counselling for PCD would take into account different genetic effects on cilia versus sperm motilities. Therefore, in this review we present the current knowledge of PCD-associated male infertility and sperm tail phenotypes caused by mutations in axonemal genes.

## Current PCD diagnostics

Primary ciliary dyskinesia is a genetically heterogeneous disorder of variable clinical impact and thus far there is no single or even combination of tests accurate enough for making a diagnosis under all circumstances [55, 72, 106, 112]. After suspecting a possible PCD phenotype from clinical history (neonatal respiratory distress, respiratory phenotypes, laterality defects), a series of tests can be used to confirm the diagnosis. Nasal nitric oxide gas is reduced in most patients with PCD [10]. Analysis of the respiratory epithelium by nasal brushing can be used for identification of ciliary structural defects by transmission electron microscopy (TEM) [113, 118] and defects of the ciliary beat pattern and frequency can be identified by high-speed video microscopy analysis (HSVMA) [107]. HSVMA has excellent sensitivity and specificity for PCD and particular beat patterns have been linked to specific ultrastructural defects [15]. Assisting analysis includes immunofluorescence to look at cilia protein defects [119]. This is becoming more routinely used, which in addition to more specialized electron tomography [116] can be used in difficult to diagnose cases.

Primary ciliary dyskinesia is caused by genetic variants in genes coding for proteins, which have a role in motile cilia structure, formation and function. Thus, genetics has become a more prominent component of the diagnostic pathway for PCD over recent years, with confirmation of PCD diagnosis now defined as being made through identification of an ultrastructural defect by transmission electron microscopy or the identification of bi-allelic mutations in a known PCD gene [72, 112]. Several ultrastructural defects have been identified in the respiratory cilia of patients with PCD, the majority involving absence, partial absence or shortening of the outer or both inner and outer dynein arms. Other defects include complete or partial absence of the central microtubular pair often accompanied by transposition of an outer microtubular doublet [13, 48, 91, 95], a variety of microtubular disarrangements associated with defects of the nexin–dynein regulator complex and inner dynein arm loss [3, 78, 138] or reduced numbers of cilia [11, 132]. A sizeable proportion of patients have no detectable ultrastructural defect, some explained by subtle defects visualized only at high resolution [23, 91, 117], or effects on the axoneme not directly affecting internal structures [9, 12]. To date, more than 40 genes with causative mutations for PCD have been identified (Table 2) and gene panels are available for genetic testing [84, 92, 136]. It is estimated that in about 70% of patients with a clinical PCD phenotype, a bi-allelic mutation in a known PCD gene can be found [105, 112]. Increased awareness of PCD and better testing mean that the average age of diagnosis is now in childhood [35] and therefore fertility assessment is rarely part of frontline PCD diagnostics, although it should be taken into account in adult PCD patients.

**Table 2:** Axonemal genes with mutations causing primary ciliary dyskinesia (PCD) and multiple morphological abnormalities of the sperm flagella (MMAF) and their effect on motile cilia and sperm tail

HGNC gene name	Functional category	Affected structure in human cilia	Associated disorder	HGNC approved name	Male infertility	Sperm structural defect (if known)	References male fertility
CCNO	Multi-ciliated epithelial cell differentiation	Reduced cilia, of normal structure	PCD (RGMC)	Cyclin O	Fertility problems suggested (*n* = 1)		[38, 89]
MCIDAS	Multi-ciliated epithelial cell differentiation	Reduced cilia, lacking motility structures	PCD (RGMC)	Multiciliate differentiation and DNA synthesis associated cell cycle protein	Not known		
CFAP298 (C21ORF59)	Axonemal dynein assembly	IDA/ODA	PCD	Chromosome 21 open reading frame 59	Not known		
CFAP300 (C11orf70)	Axonemal dynein assembly	IDA/ODA	PCD	Cilia and flagella-associated protein 300	Asthenospermia	Absence of DA	[44]
DNAAF1 (LRRC50)	Axonemal dynein assembly	IDA/ODA	PCD	Dynein axonemal assembly factor 1	Male infertility reported for 1 patient		[130]
DNAAF2 (KTU)	Axonemal dynein assembly	IDA/ODA	PCD	Dynein axonemal assembly factor 2	Asthenospermia	Abnormal DA, loss of DNAI2	[93]
DNAAF3	Axonemal dynein assembly	IDA/ODA	PCD	Dynein axonemal assembly factor 3	Infertility reported (*n* = 1)		[83]
DNAAF4 (DYX1C1)	Axonemal Dynein assembly	IDA/ODA	PCD	Dynein axonemal assembly factor 4	Infertility reported		[125]
DNAAF5 (HEATR2)	Axonemal dynein assembly	IDA/ODA	PCD	Dynein axonemal assembly factor 5	Not known		[21]
LRRC6	Axonemal dynein assembly	IDA/ODA	PCD	Leucine-rich repeat containing 6	Asthenospermia	Absence of DA	[54, 65]
PIH1D3 (DNAAF6)	Axonemal dynein assembly	IDA/ODA	PCD	PIH1 domain containing 3	Asthenospermia	Absence of DA	[92, 98]
SPAG1	Axonemal dynein assembly	IDA/ODA	PCD	Sperm-associated antigen 1	Infertility (*n* = 1)		[88, 130]
ZMYND10 (DNAAF7)	Axonemal dynein assembly	IDA/ODA	PCD	Zinc finger MYND-type containing 10	Infertility	Absence of DA	[16, 85, 97]
CCDC103	Axonemal dynein complex (ODA)	IDA/ODA	PCD	Coiled-coil domain containing 103	Infertility, variable	Absence of DA	[99, 100]
ARMC4	Axonemal dynein complex (ODA)	ODA docking/attachment	PCD	Armadillo repeat containing 4	Not known		[18]
CCDC114	Axonemal dynein complex (ODA)	ODA docking/attachment	PCD	Coiled-coil domain containing 114	Fertility not affected		[94]
CCDC151	Axonemal dynein complex (ODA)	ODA docking/attachment	PCD	Coiled-coil domain containing 151	Not known		
DNAH11	axonemal dynein complex (ODA)	ODA (visible by electron tomography)	PCD	Dynein axonemal heavy chain 11	Infertility (*n* = 4), fertility (*n* = 3)		[71, 130, 147]
DNAH17	Axonemal dynein complex (ODA)		MMAF	Dynein axonemal heavy chain 17	Asthenoteratospermia	Absence of CP and ODA	[137]
DNAH5	Axonemal dynein complex (ODA)	ODA	PCD	Dynein axonemal heavy chain 5	Infertility (*n* = 1), fertility (*n* = 3)		[33, 129]
DNAH9	Axonemal dynein complex (ODA)	ODA	PCD	Dynein axonemal heavy chain 9	Not known		
DNAI1	Axonemal dynein complex (ODA)	ODA	PCD	Dynein axonemal intermediate chain 1	Infertility (*n* = 3)		[130]
DNAI2	Axonemal dynein complex (ODA)	ODA	PCD	Dynein axonemal intermediate chain 2	Infertility in one male reported		[69]
DNAL1	Axonemal dynein complex (ODA)	ODA	PCD	Dynein axonemal light chain 1	Not known		
MNS1	Axonemal dynein complex (ODA)	ODA docking/attachment	Mild PCD, situs inversus	Meiosis specific nuclear structural 1	Infertility suggested		[126, 146]
NME8	Axonemal dynein complex (ODA)	ODA	PCD	NME/NM23 family member 8	Not known		[82, 108]
TTC25	Axonemal dynein complex (ODA)	ODA docking/attachment	PCD	Tetratricopeptide repeat domain 25	Not known		
DNAH6	Axonemal dynein complex (IDA)	CP	Candidate PCD^a^	Dynein axonemal heavy chain 6	Associated with globozoospermia	Head defect	[59]
DNAH1	Axonemal dynein complex (IDA)	IDA/CP	Candidate PCD^a^, MMAF	Dynein axonemal heavy chain 1	Asthenoteratospermia	Absence of CP and IDA	[2, 6, 19, 135]
DNAH2	Axonemal dynein complex (IDA)		MMAF	Dynein axonemal heavy chain 2	Asthenoteratospermia	Absence of IDA	[63]
CCDC39	IDA and microtubular organization	MT disorganization/IDA	PCD	Coiled-coil domain containing 39	Oligoasthenospermia	Axonemal disorganization	[8, 78]
CCDC40	IDA and microtubular organization	MT disorganization/IDA	PCD	Coiled-coil domain containing 40	Asthenospermia	Axonemal disorganization	[8, 140]
CCDC65 (DRC2)	Nexin–dynein regulatory complex	N-DRC	PCD	Coiled-coil domain containing 65	Not known		[145]
DRC1	Nexin–dynein regulatory complex	N-DRC	PCD	Dynein regulatory complex subunit 1	Not known		
GAS8	Nexin–dynein regulatory complex	N-DRC	PCD	Growth arrest specific 8	Asthenospermia		[49]
DNAJB13	Radial spoke complex	RS/CP	PCD	dnaJ heat shock protein family (Hsp40) member B13	Oligoasthenospermia (*n* = 1)		[27, 37, 62]
RSPH1	Radial spoke complex	RS/CP	PCD	Radial spoke head 1 homolog	Infertility reported (*n* = 1)		[130]
RSPH3	Radial spoke complex	RS/CP	PCD	Radial spoke 3 homolog	infertility reported (*n* = 1)		[48]
RSPH4A	Radial spoke complex	RS/CP	PCD	Radial spoke head 4 homolog A	fertility reported (*n* = 3)		[130]
RSPH9	Radial spoke complex	RS/CP	PCD	Radial spoke head 9 homolog	Not known		[13]
WDR66 (CFAP251)	Radial spoke complex		MMAF	Cilia and flagella-associated protein 251	Asthenoteratospermia	Absence of CP, disorganized axoneme, FS, ODFs and MS	[4, 51, 60, 61]
STK36	Radial spoke/central pair connection	CP	PCD	Serine/threonine protein kinase 36	Not known		[26]
HYDIN	Central pair complex	CP (visible by electron tomography)	PCD	HYDIN, axonemal central pair apparatus protein	Asthenoteratospermia	Rigid/immotile sperm	[91]
AK7	Central pair complex		MMAF	Adenylate kinase 7	Asthenoteratospermia	Absence of CP	[70]
ARMC2	Central pair complex		MMAF	Armadillo Repeat Containing 2	Asthenoteratospermia	Absence of CP	[18]
CEP135	Central pair complex		MMAF	Centrosomal protein of 135 kDa	Asthenoteratospermia	Short or absent tail (no EM)	[111]
CFAP43	Central pair complex		MMAF	Cilia- and flagella-associated protein 43	Asthenoteratospermia	Misaligned, missing CP	[19]
CFAP44	Central pair complex		MMAF	Cilia- and flagella-associated protein 44	asthenoteratospermia	disassembled, missing CP	[19]
CFAP69	Central pair complex		MMAF	Cilia- and flagella-associated protein 69	Asthenoteratospermia	Absence of CP	[22, 40]
QRICH2	Central pair complex		MMAF	Glutamine-rich protein 2	Asthenoteratospermia	Absence of CP, disorganized axoneme and ODFs	[114]
GAS2L2	Ciliary orientation	Normal structure, abnormal orientation	PCD	Growth arrest-specific protein 2-like 2	Not known		[12]
LRRC56	IFT, distal tip dynein assembly	Normal in human but linked to distal ODA	Candidate PCD^a^	Leucine-rich repeat domain-containing protein 56	Not known		[9]
SPEF2	IFT		MMAF	Sperm flagellar 2	Asthenoteratospermia	Absence of CP, disorganized accessory structures	[64, 67]
TTC21A	IFT		MMAF	Tetratricopeptide repeat domain 21A	Asthenoteratospermia	Absence of CP, missing or disorganized axoneme, malformed HTCA	[66]

*HGNC* Human Gene Nomenclature Committee

^a^Referred to as such where either the gene variant data or cilia structure/function evidence is insufficient to prove definitive PCD

## How do motile cilia and sperm differ?

The gross axonemal structure of the motile cilia and sperm tail may appear to be identical, but cell type-specific differences in axonemal proteins such as dynein arm components and in assembly of the axoneme exist [32]. This conclusion is supported by the fact that basic motility and aspects of morphology differ between cilia and sperm and also since not all mutations in PCD genes cause male infertility (Table 2). Differences exist in the length of the sperm flagellum and in the accessory structures surrounding the axoneme compared to cilia. This is linked to the distinct motility pattern and functions of motile cilia versus sperm tails. Cilia motility includes a forward power stroke to move the mucus in airway epithelia and a recovery return stroke to then produce another power stroke [123], but in sperm the bending waves of the tail produce a constant forward pushing motion [47]. The respiratory tract cilia are attached to the apical surfaces of the airway epithelium moving the overlying fluid, whereas the flagellum of spermatozoa has evolved to power movement of the gamete freely through fluid.

Because of the role of motile cilia in mucus removal in the airways, multiciliogenesis is required for production of multiple cilia in the epithelial cells [17]. This differentiation programme is not necessary for sperm tail formation [58] or laterality determination [39] and thus mutations in genes encoding proteins with a role in multiciliogenesis (e.g. CCNO and MCIDAS) are not expected to affect male germ cell development. However, recent studies indicate that multiciliogenesis is required for correct function of the efferent ductules [20, 142]. In mice it has been shown that mutations in multiciliogenesis genes, *Gemc1*, *Mcidas* and *Ccno*, cause male infertility and complete lack of sperm in the epididymis [127].

Conversely, defects in sperm tail-specific structures can cause infertility with no airway phenotype. The long axoneme within the sperm flagellum is supported by the ODFs and FS and therefore malformations in these structures may also cause instability of the axonemal structure, as has been seen in patients with mutations in the FS protein, FSIP2 (fibrous sheath interacting protein 2) [28]. Depletion of another FS protein, AKAP4 (A-kinase anchoring protein 4), gives rise to lowered sperm numbers and short sperm tails with disruptions to the principal piece formation of the sperm tail (asthenoteratospermia), but the axoneme appears intact [5, 79]. Male infertility has also been reported to be caused by mutations in outer dense fibre gene *ODF1*, where the head tail connection was weakened in addition to disorganization of the ODFs and MS [41, 139]. Sperm tail-specific proteins may also be involved in attachment of the axoneme to the ODFs and FS [19] and unidentified or poorly characterized differences affecting the structural components may also contribute to the specific requirements of the sperm tail. This has been demonstrated by the identification of the tail axoneme intra-lumenal spiral (TAILS), a structure which binds directly to 11 protofilaments on the internal microtubule wall, along the end piece of the sperm tail [70, 143].

## Differences in tissue distribution of dynein arm genes and the effect of known mutations

There is evidence that different motility patterns of cilia and sperm may require cell specific dynein arm complexes. In respiratory cilia mutations in ODA genes affect the ciliary beating and ODA ultrastructure, but no mutations in IDA genes have been identified in PCD patients to date. ODAs and IDAs are required for motility in both cilia and sperm, but differences in protein content of these complexes may contribute to the specialized motility-specific patterns required for forward swimming sperm, cilia mucus clearance in airways and the rotational movement of nodal cilia. In 9 + 0 nodal cilia, their lack of central pair microtubules and radial spokes seems to be the key for their unidirectional rotational movement [115], but the structural differences explaining the differences in motility between 9 + 2 cilia and sperm are not immediately obvious.

**Fig. 2:**
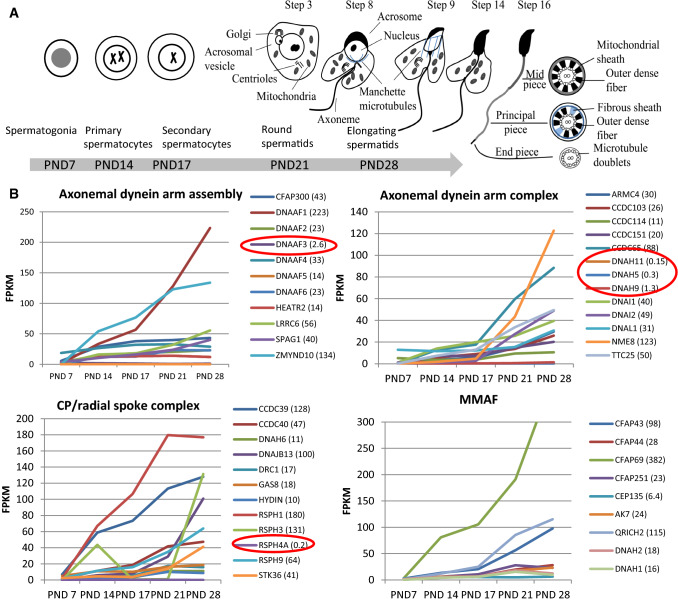
PCD gene expression during the first wave of mouse spermatogenesis. **a** Cell type appearance of mouse sperm during the first wave of spermatogenesis. In addition to specific populations of male germ cells, all samples contain somatic cells. *PND* post-natal day. **b** PCD gene expression changes during the progression of mouse spermatogenesis, divided up according to the different functional categories of PCD genes. RNAseq was conducted on testis tissue samples collected at specific postnatal time points as indicated in 3A [56]. Red circles indicate the detected levels of genes *Dnah5*, *Dnah9*, *Dnah11* and *Rsph4a*, showing their very low levels of expression as discussed in the text, which supports a potentially lesser role in sperm compared to their known motile cilia-specific functions. In the mouse testis, *Dnaaf3* expression was also limited. All identified MMAF genes show a pattern of increasing expression during the progression of spermatogenesis. The highest reads per kilobase million (RPKM) expression value is indicated in brackets

**Fig. 3:**
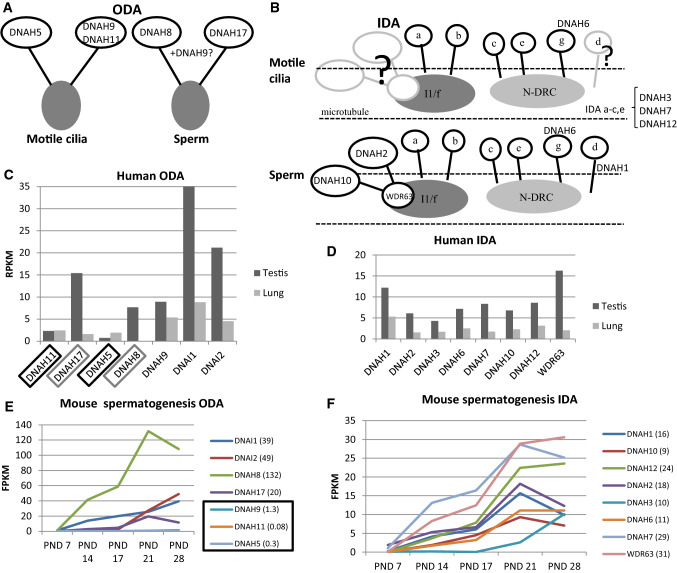
Expression of axonemal dynein arm genes in motile cilia and during spermatogenesis. A model of suggested differences in components of ODA (**a**) and IDA (**b**) in motile cilia versus sperm, based on evidence of expression studies from Drosophila [128], human [26] and mouse [50]. The IDA is a suggested model, because in humans there are approximately 7 isoforms [1 double headed dynein (I1/f) and 6 single dynein IDA complexes (a–e, g)] which are thought to be structurally different within an axonemal 96-nm repeating pattern [104]. The human IDA structure is much less resolved in terms of its possible subunit components and more complicated than the human ODA. ODA (**c**) and IDA (**d**) DNAH gene expression in the human testis and lung [26]. ODA component DNAH8 is not expressed in the lung and DNAH17 shows low expression (grey box). In contrast, DNAH5 and DNAH11 have low expression in the testis (black box) compared to the lung. DNAH2, DNAH10 and DNAH binding gene WDR63 show low transcript levels in the lung, but DNAH1 expression in relatively high compared to axonemal gene expression in general. *RPKM* reads per kilobase million. ODA (**e**) and IDA (**f**) gene expression during the first wave of mouse spermatogenesis (see Fig. 2 for graph of cell content during the first wave of spermatogenesis in mouse). Dnah5, Dnah9 and Dnah11 show very low expression during mouse spermatogenesis [black box, fragments per kilobase million (FPKM) expression levels indicated in brackets]. All IDA genes show high expression at PND21 during the axoneme formation, except Dnah3 may be required later during the spermatid elongation

Recent expression studies in Drosophila indicate that specific ODA and IDA components are required for different waveforms in sperm and neuronal cilia [148]. Previously published transcriptome profiles during the first wave of spermatogenesis in mice [56] show the PCD gene expression pattern during male germ cell development. This dataset consist of testis tissue samples at five different postnatal (PND) time points, which contain specific male germ cell populations (Fig. 2a). Furthermore, analysis of RNAseq data from Fagerberg et al. [29] suggests that the cilia ODA dynein axonemal heavy chain (DNAH) proteins DNAH5 and DNAH11 may be replaced in sperm by DNAH8 and DNAH17 (Fig. 3a). This is supported by sperm-specific symptoms in patients with mutations in *DNAH17* [137]. *DNAH8* is not expressed in the lung and low level of expression is detected for *DNAH17* (Fig. 3b). Very low levels of expression are detected for *DNAH5* and *DNAH11* in the human testis, but *DNAH9* appeared present (Fig. 3B). PCD mutations in *DNAH9* have recently been identified and for one patient very low sperm motility (5% normal progressive motility) was reported [31, 68]. This suggests that DNAH9 may have a role in sperm motility. During mouse spermatogenesis *Dnah5, Dnah9* and *Dnah11* expression was extremely low (Fig. 3c), indicating that there may also be species specific differences. Similar results have been reported for testis-specific expression on RNA and proteomic levels of DNAH8 and DNAH17 and motile cilia-specific expression of DNAH5, DNAH9 and DNAH11 [137].

**Fig. 4:**
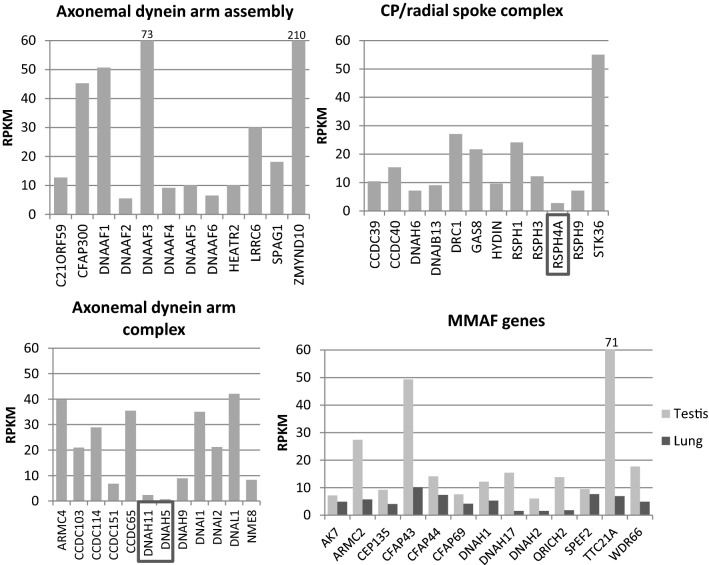
PCD gene expression in the human testis and MMAF gene expression in the lung. PCD genes are divided up into functional categories in each graph. *DNAH5*, *DNAH11* and *RSPH4A* show markedly low expression (RPKM < 3) in the testis (boxes) compared to other PCD genes. The high RPKM value for *DNAAF3*, *ZMYND10* and *TTC21A* is indicated above the bar (73, 210 and 71, respectively). Some expression is also detected for MMAF genes in the lung. The data was produced by RNAseq of human tissues samples [29]

Immunofluorescence results from PCD patients with mutations in ODA genes show a lack of DNAH9 along the cilium [33, 69, 81], but DNAH9 was reported to be present in sperm from patients with *DNAH5* and *DNAL1* (dynein axonemal light chain 1) mutations [33]. This result suggests that DNAH5 or DNAL1 are not required for DNAH9 localization along the sperm tail, although DNAL1 is highly expressed in the testes (Fig. 4). *DNAL1* mutations are a very rare cause of PCD [77], but male infertility has been identified in a patient with a mutation in the dynein axonemal intermediate chain gene (*DNAI2*), which was also missing from the sperm tail in a male PCD patient with mutations in dynein axonemal assembly factor 2 (*DNAAF2*) [93].

In general, a limited amount of information is available about the effects of dynein arm mutations on male fertility. Amongst the most common PCD-causing mutations are those affecting the ODA genes *DNAH5* and *DNAH11* [45, 144]. The effect of *DNAH5* mutations on male fertility has been reported in a small number of individuals, but with contradictory results. Oligo- and azoospermia was reported for two men with *DNAH5* mutations [33] and fertility reported for three men and infertility for one, in a separate study [129]. In oligo- and azoospermic patients, localization of DNAH5 along the sperm tail was reported in patient and control sperm, although it was reduced/absent in the cilia [33]. Since a lack of staining of sperm DNAH5 was previously published [137], we suspect that the identified DNAH5 staining along the sperm tail [33] is unspecific. In *Dnahc11*^*iv*^ mice homozygous for a Glu2271Lys missense mutation in *Dnah11*, there are defects in sperm motility, but without fertility being affected [71]. The *Dnahc11*^*iv*^ sperm axonemal ultrastructure was also undisturbed in TEM analysis, however it is known that in cilia *DNAH11* mutations confer a very subtle ultrastructure defect to the ODAs which so far can only be visualized using the much higher resolution of 3D tomography [23, 117]. In agreement with these variable outcomes, several male patients with *DNAH11* mutations have been reported to father children without assisted techniques, whereas others have been diagnosed as infertile [109, 129]. Thus, it appears that complete function of DNAH5 and DNAH11 is not always crucial for male fertility.

In addition to ODA components, there might be differences in IDA proteins between motile cilia and sperm. This has been suggested by studies in Drosophila, where the IDA proteins DNAH2, DNAH10, WDR63 (WD repeat domain 63) and CASC1 (cancer susceptibility 1) appear to be testis specific [148] and some indication of low expression of *DNAH2, DNAH10* and *WDR63* was also seen in the transcriptomic data of the human lung (Fig. 3b, [29]). However, identification of the role of different proteins of the ODA and IDA in human motile cilia versus sperm requires further studies. Interestingly, although no mutations in IDA genes have yet been identified for PCD, a sperm-specific phenotype called multiple morphological abnormalities of flagella (MMAF), where sperms are severely malformed (teratozoospermia), has been reported in affected individuals carrying mutations in *DNAH1* [2, 6, 19, 135] and *DNAH2* [63].

## The effect of PCD mutations in dynein arm assembly genes and structural components on sperm development

Apart from dyneins, mutations in a number of other structural proteins of the axoneme also cause PCD, including mutations in CCDC65, SPAG1 (sperm-associated antigen 1) and NME8 (NME/NM23 family member 8) which have all been localized along the sperm tail [82, 88, 108, 145]. Axonemal disorganization was also described in sperm from patients with PCD-causing mutations in the ‘ruler genes’ *CCDC39* and *CCDC40* [90], which is a comparable phenotype to that of the patient’s respiratory multiple motile cilia. Subtle ODA defects have been reported in patients with mutations in *MNS1* (meiosis specific nuclear structural 1), accompanied by male infertility, situs inversus and mild PCD symptoms [126]. MNS1 is an axonemal protein found to dimerize and interact with the ODA docking complex component CCDC114, which is also mutated in PCD [94, 126, 133]. Based on previous publications, fertility is not affected in PCD patients with *CCDC114* mutations [94] and the expression level is low in the mouse, but relatively high in the human testis. CCDC114 is therefore probably not crucial for sperm function, or as previously proposed, it can be compensated by other gene products such as CCDC63 during spermatogenesis [94]. Mutations in several central pair complex and radial spoke head proteins have been identified in PCD patients. For patients with mutations in the C2 central pair projection gene *HYDIN*, infertility has been reported in affected men [59, 91]. Subfertility caused by sperm motility defects have also been reported for male PCD patients with mutations in the radial spoke genes *DNAJB13* (DnaJ heat shock protein family (Hsp40) member B13), *RSPH3* (radial spoke head 3 homolog) and *RSPH9* [13, 27, 37, 48, 62]. Interestingly, the PCD protein RSPH4A seems to be dispensable for spermatogenesis, since male PCD patients with *RSPH4A* mutations have been reported to be fertile and its expression levels in the testis are very low ([130], Fig. 4). These contrasting effects on male fertility suggest that differences between sperm flagella and cilia exist for the radial spoke head proteins, since mutations in any of the RSPH genes seems to have a similar consequence in the cilia; central pair loss and motility defects. A compensatory gene for RSPH4A in the testis may be RSPH6A, which has not been associated with PCD, but depletion of the mouse *Rsph6a* is a reported cause of male infertility [1].

Mutations in axonemal dynein complex preassembly genes have often been reported to cause male infertility in PCD (Table 2). In addition to mutations in *DNAAF2* [93], PCD-causing mutations in *DNAAF1, DNAAF3* and *DNAAF4* have been reported to cause male infertility, but the patient number is minimal (Table 2). Mutations in their interacting partner, the dynein complex assembly factor PIH1 domain containing 3 (*PIH1D3*) have also been shown to result in lack of IDA and ODA [92, 98]. Male infertility has also been detected in PCD patients with mutations in axonemal dynein assembly factors *LRRC6* (leucine-rich repeat containing 6), *ZMYND10* (zinc finger MYND-type containing 10) and cilia and flagella-associated protein 300 (CFAP300). PCD with a lack of dynein arms in both the cilia and sperm is reported for *LRRC6, ZMYND10, CFAP300* and axonemal dynein complex gene *CCDC103* (coiled-coil domain containing 103) [16, 44, 54, 65, 97, 99, 100]. However, for *CCDC103* mutations variable male fertility phenotype has been reported; one patient showed a lack of DA and DRC comparable to the motile cilia phenotype, but the other was fertile without DA loss [100].

## Genetic mutations causing sperm tail-specific phenotypes

Mutations in a number of human genes result in sperm motility defects of a structural nature that is within the same spectrum as those caused by mutations in PCD genes. In these cases, associated with malformation of the sperm tail and low sperm count, mutations in a number of genes not connected to the respiratory PCD phenotype are reported. These apparently only affect the sperm tail axoneme and not cilia functions, having been identified as the cause of MMAF. The main findings in MMAF are absent, short, bent, coiled, and/or irregular sperm flagella with very low numbers of normal spermatozoa (0–2%) associated with low sperm concentrations [6]. A MMAF sperm tail phenotype of short, stumpy tails has also previously been described as dysplasia of the fibrous sheath (DFS) [5, 14, 102]. DFS has been reported to be caused by mutations in three genes encoding components of the sperm tail specific fibrous sheath: *AKAP3*, *AKAP4* and *FSIP2* [5, 75]. AKAP3 and 4 are the main components of the fibrous sheath and FSIP2 is required for localization of AKAP4 to the FS [75].

Sperm tail accessory structures are also often disorganized in MMAF patients with mutations in axonemal genes, such as *DNAH1*, which is the most common identified cause of MMAF [2, 6, 135]. DNAH1 is a component of the IDAs, which are completely disorganized in sperm tail axonemes of MMAF patients with *DNAH1* mutations [6]. Mutations in another IDA gene, *DNAH2*, have also been identified as a cause for MMAF [63]. The sperm tail phenotype is similar in patients with *DNAH1* and *DNAH2* mutations with lack of the CP or complete disorganization of axonemal and ODF structures in addition to IDA loss [2, 6, 63, 135] indicating that IDAs are crucial for sperm tail axoneme integrity. Infertility has been the only PCD symptom identified in MMAF affected men with *DNAH1* and *DNAH2* mutations suggesting that these IDA components are not required for motile cilia function. However, *DNAH1* mutations have also been reported in one family with siblings affected by PCD [46] and *DNAH1* appears to be expressed in the lung (Fig. 3B). The possible role of *DNAH1* and other IDA genes in PCD awaits to be revealed. IDA gene mutations may cause milder PCD symptoms due to the role of IDAs in controlling the wave form [52].

There seem to be differences in the severity of the sperm tail phenotype caused by IDA mutations between species in addition to differences between motile cilia and sperm. *Dnah1* knock-out (KO) mice only show motility, but no structural sperm defects (asthenozoospermia) [87], whereas in humans *DNAH1* truncating mutations induce a MMAF phenotype [2, 135]. Decreased cilia beat frequency was also detected in the mutant mice, which suggests a comparable role of DNAH1 in mouse sperm tail and motile cilia. In humans the lack of DNAH1 in motile cilia could be compensated by other DNAH genes such as DNAH12, which is the closest paralog to DNAH1 [74].

A report on the effect of sperm-specific ODA heavy chain DNAH17 mutations was recently published [137]. DNAH17 mutations were identified in five MMAF patients with sperm lacking ODAs. Some sperm tail cross sections showed missing CP and even disorganized outer doublet microtubules, but no defects were seen in the patient’s respiratory cilia. Thus, DNAH17 mutations only appear to affect sperm tail ODA formation, a finding also supported by expression and protein localization studies [137].

The cilia and flagella-associated protein (CFAP) CFAP251, also known as WDR66, a component of the calmodulin- and radial-spoke-associated complex [24, 25] is located adjacent to DNAH1 in *T. thermophila* at the base of the RS3 [42, 53] radial spoke complex. The amount of DNAH1 homolog IDA g was reduced in *T. thermophila* CFAP251 KO [128] and a direct physical link between CFAP251, IDA3, and RS3 has been shown by co-immunoprecipitation experiments [4]. Mutations in *WDR66* cause a typical MMAF phenotype with immotile, malformed sperm tails in which all the sperm tail structures are disorganized [4, 51, 61].

The main axonemal ultrastructural finding in MMAF patient sperm seems to be the lack of central pairs from the 9 + 2 microtubular arrangement (Table 2). Mutations in *ARMC2* (armadillo repeat containing 2), *CFAP43* and *CFAP44* cause male infertility associated with sperm axonemal central pair defects [18, 19]. The main defect based on the human sperm EM appears to be the lack of central pair, although some completely disorganized axonemal and accessory structures were also observed. The *Armc2* KO mouse model shows a similar phenotype and immunofluoresence experiments indicated the presence of other axonemal components except the central pair [18]. In patients with *CFAP43* and *CFAP44* mutations, some variable axonemal phenotypes were also observed [19]. In some sperm tail cross sections, the missing central pair was accompanied by disorganized outer doublets and in patients with *CFAP43* mutations the central pair was misoriented when present. In KO mouse models, mutations in *Cfap43* showed a more dramatic sperm tail phenotype than for the *Cfap44* KO. Only short disorganized tails were detected in *Cfap43* mutant mice while *Cfap44* KO showed normal flagellar length, but with irregular mid-pieces. Another study reported short and immotile tail phenotypes arising from mutations in both genes [124]. CFAP43 and CFAP44 are suggested to have a role in connecting specific doublet microtubules to periaxonemal structures in the sperm tail and thus may not be required for motile cilia. The trypanosome ciliate homologs were localized on the outer surface of the doublet microtubules [19].

Mutations in *CFAP69* were reported in two MMAF patients with typical short immotile sperm tail characteristics that were replicated in *Cfap69* KO mice, thereby confirming a role for CFAP69 in spermiogenesis [22]. In mature human sperm, CFAP69 is localized in the mid-piece, thus it may not be a structural component of the axoneme. However, all sperm tail structures were completely disorganized in the *Cfap69* mutant mouse model. Human patients with *CFAP69* mutations have a variable sperm tail phenotype depending on their mutation. Sperm-specific phenotypes have also been suggested for mutations in *CEP135* (centrosomal protein 135) and *AK7* (adenylate kinase 7). In one MMAF patient with short or absent sperm tails, a missense mutation in *CEP135* was identified as a probable cause for infertility [111]. A sperm-specific phenotype with incomplete mitochondrial sheath, dysplasia of the fibrous sheath and lack of central pair microtubules with axonemal disorganization was also reported for *AK7* [70]. PCD-associated mutations and decreased AK7 expression have also been reported in PCD patients [76, 80]. Tissue specific effects of *AK7* mutations are likely, since a missense mutation was reported to affect the expression in the testis, but not in multiciliated tissues [70].

Recently, mutations in *SPEF2* (sperm flagellar 2) have been reported to cause MMAF and interestingly some of the patients show mild PCD-like symptoms [64, 67]. Variable *SPEF2* mutant phenotypes have also been demonstrated in animal models, where depletion of different gene variants causes sperm-specific or PCD-like symptoms [57, 121, 122]. The variability of symptoms can potentially be explained by the nature of the gene variants and severity of their effect on protein function and/or the nature of the proteins themselves. It has been suggested that SPEF2 has a role in protein transport and it interacts with IFT20 (intraflagellar transport 20) [120]. Another IFT-associated protein TTC21A (tetratricopeptide repeat domain 21A) was also reported to cause MMAF with malformed sperm tails and additional abnormalities in the sperm HTCA [66]. Thus, sperm or motile cilia-specific IFT-associated proteins may also be required for transport of proteins in these specialized cilia types.

For most MMAF patients, no PCD-associated respiratory symptoms have been identified. Associated motile cilia defects and disease symptoms if any could be mild, variable and hard to detect in some cases. However, phenotypes rather tend to suggest that roles of at least some of the identified gene products are potentially restricted to the male germ cells. This could have something to do with involvement in stabilizing the long sperm tail axoneme or with connection of the axoneme to the accessory structures. In addition, it seems clear that different dynein arm proteins can be more crucial for sperm tail functions than for motile cilia. Thus, the different motion patterns of cilia and sperm may require specific protein components. These differences require further investigation in larger numbers of patients in order to elucidate the exact differences between human cilia and sperm tail formation. Most mutations for MMAF have been identified in North African and Chinese populations and a few single French patients, hence human data is sparse.

## Clinical implications of sperm defects in PCD and fertility treatment

Male fertility can be affected by many genetic and non-genetic factors, but infertility arising from axonemal defects is a clearly testable phenotype [30]. For PCD patients presenting at respiratory clinics, age-appropriate genetic and fertility counselling should be available or in place. Arrangements for fertility testing should be made available if required and this is especially important around the time of transition from paediatric care to the adult clinic in PCD males. Knowledge is increasing with regards to which PCD genes may or may not be associated with sperm defects and male infertility (Table 2). Currently, due to preserved sperm function in some cases of PCD, the fertility phenotype should be assessed by laboratory confirmation alongside or ideally prior to counselling of patients. Knowledge of male infertility phenotype/genotype correlations is the prerequisite for the most appropriate counselling of patients, based on their result from genetic testing for PCD.

The diagnostics and family counselling of PCD should be informed by the genetic background of the defect. Indeed, PCD patients seeking in vitro fertilization (IVF) privately without knowledge of their condition and the effect on sperm motility may face the problem of sperm viability (related to motility) being a prerequisite for being sent forward for IVF; they will benefit from the fullest knowledge around their condition. Thus far, an estimated 70% of PCD patients can be expected to have mutations in known causative genes, but the effect of these mutations on male fertility is largely unknown. Future studies will establish novel PCD mutations, which can be assisted by identification of teratozoospermic or asthenozoospermic patients, who may have mild PCD symptoms. In rare cases azoospermia has also been reported for PCD patients, thus these patients may also have mutations in cilia/flagella related genes. Although some implications of the effect of PCD mutations on male infertility have emerged, the comprehensive analysis of association of sperm phenotype with specific PCD mutations requires future studies. On the other hand, identification of the effects on male fertility of specific PCD mutations in specific genes is of great importance and enables the inclusion of PCD genes on wider gene panels for male infertility.

For those PCD patients who are not able to conceive naturally, several studies report successful intracytoplasmic sperm injection (ICSI) based fertilization of eggs and consequent IVF births with sperm from PCD patients. Successful ICSI outcomes of PCD sperm have been reported for 28% of cases with live births [50], although the fertilization rate was much higher, at 80%. The low live birth rate compared to fertilization rate may imply developmental problems during embryogenesis. This may be due to poor embryo quality, which could result from functional failure of the sperm-derived centrosome. Centrosome dysfunction often leads to irregular cleavage during embryo development or chromosomal aberrations, resulting in delays or arrest of embryo development [103]. The specific mutations within the PCD cases were not reported, thus the effect of the genetic cause on ICSI outcome requires further studies. Reports for MMAF patients show that spermatozoa with mutations in the centrosomal gene *CEP135* produced no pregnancy after embryo transplantation [111]. However, the ICSI outcome for MMAF patient sperm from *DNAH1* mutation carriers shows good prognoses, possibly due to these mutations primarily affecting the structure of the sperm axonemal inner dynein arms [134]. Successful ICSI outcomes were also shown for *CFAP43* and *CFAP44* patients [110]. Outcomes of ICSI are reported to be improved through testicular sperm extraction and with the addition of pentoxifylline treatment [34, 141]. Further studies on the effects of specific gene mutations on male fertility and ICSI outcome are required to improve the diagnosis and treatment of male infertility in PCD patients.

## Conclusion

Although it appears that many PCD genes have an effect on male fertility, it is clear that the comparative disease phenotype between cilia and sperm does not correlate in all cases. For example, DNAH11 and DNAH5 may be cilia specific and DNAH17 and DNAH8 have a role only in the sperm axoneme, with consequent cilia- and sperm-restricted defects seen in affected patients. Thus far, male infertility specific phenotype (MMAF) has been reported for mutations in 13 potentially axonemal genes, *DNAH1, DNAH2, DNAH17, CEP135, WDR66, SPEF2, ARMC2, TTC21A, QRICH2* and *CFAP* genes *43, 44, 69* and *251*. Expression of some of these genes is limited to the testis, however some may also have roles in motile cilia as suggested for SPEF2 [64, 67]. Testis- and cilia-specific compensatory genes could prevent the effect of specific mutations in spermio- or ciliogenesis, respectively, as has been speculated for CCDC114 [94]. Male infertility in PCD has been documented for 22 PCD genes (Table 2), but the patient numbers are very low and the exact effect of mutations on sperm tail structures is not known in most cases. Therefore it is of high importance to investigate the exact effect of PCD mutations on male fertility. Information on the spermatogenesis specific roles of ciliary genes will enable counselling of patients about their fertility status and possible clinical treatments required for conceiving.
